# A pilot study of disease related education and psychotherapeutic support for unresolved grief in parents of children with CF

**DOI:** 10.1038/s41598-022-09463-8

**Published:** 2022-04-06

**Authors:** André Schultz, Andrea Barrett, Elizabeth Balding, Wesley Billingham, Cindy Branch-Smith, Zubin Grover, Gisele Yikilmaz, Crystal Bourke, Julie Depiazzi, Nicole Sander, Juliet Foster, Matthew Cooper, Florian Zepf

**Affiliations:** 1 Wal-Yan Respiratory Research Centre, Telethon Kids Institute, University of Western Australia, Perth, Australia; 2 Division of Paediatrics and Child Health, Faculty of Medicine, University of Western Australia, Perth, Australia; 3 Perth Children’s Hospital, Perth, Australia; 4 Woolcock Institute of Medical Research, Sydney, Australia; 5 Department of Child and Adolescent Psychiatry, Psychosomatic Medicine and Psychotherapy, Jena University Hospital, Friedrich Schiller University Jena, Jena, Germany; 6 Centre and Discipline of Child and Adolescent Psychiatry, Psychosomatics and Psychotherapy, Division of Psychiatry & Clinical Neurosciences, School of Medicine, Faculty of Health and Medical Sciences, University of Western Australia, Perth, Australia

**Keywords:** Psychology, Cystic fibrosis

## Abstract

Diagnosis of chronic disease in a child can result in unresolved grief (UG) in parents. This study aimed to evaluate the efficacy of psychological insight-oriented therapy (IOT) as a treatment for UG compared to disease related education in parents of children with cystic fibrosis (CF). Sequence of delivery, first IOT then disease related education (or vice versa) was also examined, to let all participants experience both interventions. Parents were screened for UG. Parents with UG were randomised to either five 1-h sessions of IOT or five 1-h sessions of education. Measures were assessed pre-intervention, after the first intervention period (primary efficacy assessment), and after the second intervention period (swapping intervention). Forty-seven parents were screened of which 46.8% (22/47) had UG. Median duration of UG was 5 years (range: 6 months–14 years). Anxiety (50% vs. 20%, *p* = 0.03) and stress (59% vs. 28%, *p* = 0.03) were significantly more prevalent in parents with UG. There was no difference between arms in the odds of UG resolving either following the first intervention period (OR 0.88; 95% CI 0.5, 1.5) or the second intervention period (OR 0.91; 95% CI 0.5, 1.6). While not statistically significant, adjusted mean values for seven of the eight mental health measures were lower in the IOT (first) arm compared to the ED (first) arm, following the first intervention period. UG is a significant burden for families affected by CF. Provision of disease related education and psychological support, regardless of sequence, can result in resolution of grief.

**Trial registration number:** ACTRN12621000796886, date of registration 24/06/2021, retrospectively registered.

## Introduction

Cystic Fibrosis (CF) is an autosomal genetic and life limiting disorder that affects approximately 70,000 people^1,2^. In many countries, CF is commonly diagnosed through newborn screening^3^. The news of a CF diagnosis usually is a shock for families and coincides with an important period of laying down the early foundations for parent–child attachment^4^, thereby potentially compromising these important relational bonds.

Parents report the period directly following diagnosis to be a time of shock, disbelief, grief^5,6^, heightened emotional experiences and difficult thoughts^7^. This is also typically a time of intense CF related education and engagement with a multidisciplinary team. For some parents the initial disease related education has been found to be an overwhelming experience in which coping capacities can vary greatly^7,8^.

Parents may dissociate or push away the information about diagnosis during this particular period of crisis and struggle to reach a stage of resolution or acceptance about the diagnosis^9^. The emotional process of resolution requires an acceptance of the emotional pain and other feelings that arise with this news, as well as the acceptance of the long-term aspect of parenting a child with these challenges^9^. As the features of being unresolved are closely associated and parallel with many features of grieving, for the purposes of this study, we will refer to a lack of resolution as unresolved grief (UG).

Prolonged UG can have long-lasting effects on the quality of the parent–child relationship in terms of the parent–child attachment, and consequently on the child’s developing sense of self^10,11^. Specific and targeted interventions to address parental unresolved grief following a diagnosis of CF have not been studied to-date.

Insight oriented psychotherapy (IOT) aims to help patients develop new insights about their suffering, and to bring about positive change in a person’s internal world and state of mind. In the context of potential UG in parents of children with diagnosed CF, IOT on the parental level could be a valuable intervention for affected families.

We aimed to:

1. identify (and quantify) a lack of resolution of UG in parents, following their child’s diagnosis with CF
2. evaluate the effectiveness of IOT compared to disease related education in treating UG
3. examine the sequence of delivery of IOT followed by disease related education (or vice versa) in treating UG
4. examine the impact of IOT and ED on anxiety, depression and stress.

## Methods

### Study design

In this pilot study, participants were screened for UG. Those with UG were then randomised to a pre-post design to assess two interventions.

### Setting, participants and screening

All parents of children with CF aged 6 months–18 years and living within, or close to, the Perth (Australia) metropolitan area were identified through the only paediatric CF service in Western Australia and approached with or mailed information about the study, thus pragmatically maximising the attainable sample within this single site study. Parents who lived too far away to feasibly participate in the study’s interventions were not approached (Western Australia is very large, covering 2.646 million km^2^). In total 105 families were approached and 7 opted out of being contacted further by the study team. Parents who gave written informed-consent to participate in the study were screened for grief in relation to their child’s diagnosis with CF with the Reaction to Diagnosis Interview (RDI)^12^ administered during a 1-h interview with an RDI-trained psychologist. The RDI is explained in more detail in Online Resource 1. The interview was video recorded and later analysed by a separate investigator who was trained in the analysis of the RDI interview. Parents with UG continued to randomisation, with one parent enrolled per affected family.

### Randomisation

Following enrolment, participants were randomised to receive either IOT (IOT arm) or education (education arm). Randomisation was carried out by a third party (hospital pharmacy) using randomised blocks of four.

Following the first period of intervention, participants had a 1-to-5 week break, depending on participant availability, before moving on to the alternate intervention. The decision to give each participant the opportunity to experience both interventions was based on recommendations from clinicians in the hospital’s CF service who felt it best all participants were able to experience the IOT component of the research as parents of patients had no other means of direct access to such services through the paediatric hospital setting. Participants were assessed again for UG and emotional wellbeing following each period of intervention. Therefore, participants were assessed for UG and emotional wellbeing at three time-points pre (screening), post (after intervention one), and follow-up (after intervention two). During the course of the study, two minor changes were made to the protocol (UG eligibility criteria, age of child at diagnosis criteria); theses are further detailed in Online Resource 2.

### Interventions

#### Intervention one (insight-oriented therapy; IOT)

Comprised five 1-h one-to-one sessions of IOT with a psychologist spread out within a minimum of 5 weeks. The aim of short term IOT was to assist parents to gain a deeper understanding of the impact of their UG surrounding their child’s diagnosis on their day-to-day coping, their interpersonal relationships, and their distress surrounding the future of their child, with the aim of bringing about psychological change which would lead to a sense of resolution in relation to their child’s CF diagnosis. IOT is described more in the “Electronic Supplement”.

#### Intervention two (disease related education; hereafter ‘education’)

Comprised five 1-h sessions of CF related education spread out over a period of at least 5 weeks. Education was provided by experienced members of a CF care team including a paediatric respiratory physician, CF nurse, physiotherapist, gastroenterologist, and dietician, providing one-on-one information sessions about their areas of expertise in CF. The basic framework for each session is provided in Online Resource 3. Sessions allowed for questions from parents, and the information provided was tailored to parents’ needs. The education sessions aimed to address basic CF related information that parents may not have been receptive to at initial meetings with the clinical CF care team shortly after having been informed about the diagnosis, and that may not have been re-visited with subsequent routine clinical care visits. The overall setting of education sessions could be described as optimistic and caring.

### Outcome measures

Resolution of UG formed the primary outcome measure. Other assessed measures of emotional wellbeing included anxiety, depression, and stress, measured using the Depression, Anxiety, Stress Scale 21 (DASS^13^), and the Parent Stress Index (PSI^14^). Cut-off scores for the DASS 21 were as follows: depression ≥ 14, anxiety ≥ 10, stress ≥ 19.

### Data analysis

Descriptive statistics were calculated for the full sample and per arm. Basic between-group comparisons (at post and follow-up time-points) were made using a Chi-squared test or a Student’s t-test, as appropriate.

The primary analysis of whether UG become resolved, was assessed using a logistic regression model, with an odds ratio (OR) and 95% confidence interval (95% CI) reported for the odds of grief resolving in the IOT arm relative to the ED arm. For the other outcomes (emotional wellbeing measures), data from the end of the first period (post) were analysed using a linear regression ANCOVA framework, where the post-time-point measurement was the dependent variable and the pre-time-point measurement was included as an independent variable in the model. All regression models were adjusted for gender of the parent, age of the parent, duration of grief, and, where appropriate the initial measurement of the dependent variable for the period of analysis; minimally adjusted models, with only the initial measurement of the dependent variable for the period of analysis included, are also reported.

The secondary analysis of the follow-up time-point data were carried out via two different approaches; firstly, via the same framework as for the primary outcome using only data from the post- and follow-up time-points in place of the pre- and post-time-points (respectively) and, secondly, using data from the pre- and follow-up time-points using a ‘sequence’ variable to quantify overall change based on sequence of interventions. Model fit was accessed by visually examining residual (error) diagnostic plots to ensure regression assumptions had not be violated.

Because of the exploratory nature of this pilot study, the analysis does not focus on the statistical significance of the between arm (intervention) differences and instead focuses on the magnitude of effects and variability; *p*-values are presented for some comparisons as a further quantification of the observations against the underlying hypothesis of no difference between arms. All analyses were carried out in using a reproducible research framework implemented within R^15^.

### Ethics

The study as evaluated and approved by the West Australian Child and Adolescent Health Service Research Ethics Committee RGS2520 and conducted in accordance with relevant guidelines and regulations.

## Results

**Figure 1:**
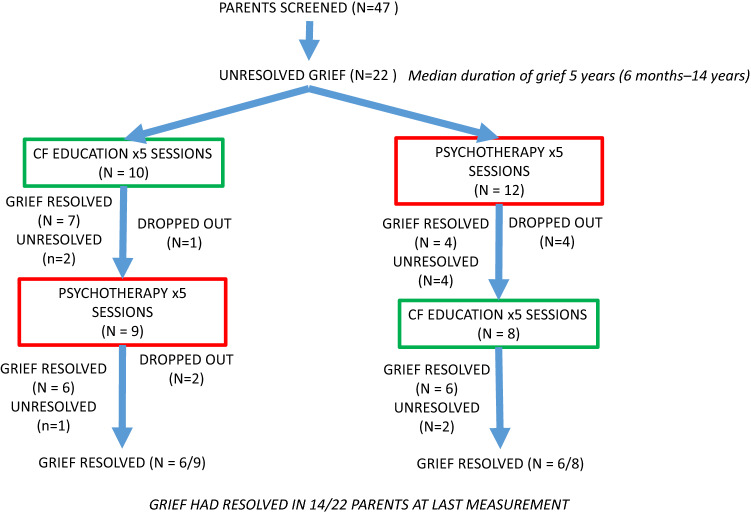
CONSORT diagram.

The CONSORT diagram is presented in Fig. 1. Briefly, 47 parents (42 female) were screened for eligibility. Anxiety was present in 34% (16/47), depression in 28% (15/47), and stress in 40% (21/47). Of the 47, 22 (48%) had UG and were randomised. For these 22, the median duration of UG was 5 years (range 6 months–14 years). Compared to the parents without UG, parents with UG had higher levels of anxiety (50% vs. 20%, *p* = 0.03) and stress (59% vs. 28%, *p* = 0.03). No difference was observed for depression. The remaining results relate to the 22 participants that were randomised, of which twelve were randomised to IOT and ten were randomised to education. Cohort descriptive statistics, by arm, are presented in Table 1; the mean age of parents was 37.4 years, and the parents were predominantly female (19, 86.3%).

**Table 1:** Cohort descriptive statistics.

	IOT arm^a^	Education arm^a^	Full sample
n	12	10	22
Age of parents in years median (range)	36.3 (6.8)	38.5 (7.9)	37.4 (7.2)
Gender (female:male)	10:2	9:1	19:3
Duration of grief	5.2 (4.4)	5.3 (4.8)	5.3 (4.5)

*IOT* insight oriented psychotherapy.

^a^Mean (sd), or count.

### Primary outcome—unresolved grief

In the IOT arm, UG became resolved in four (of the 12) parents and remained unresolved in four parents; four parents dropped out of the study before completion of this period of the study. In the education arm, UG resolved in seven (of the 10) parents and remained unresolved in two parents; one parent dropped out of the study before completion of this period of the study. In the primary outcome analysis, those in the IOT arm (relative to those in the education arm) had a non-significant, reduced adjusted odds (OR 0.88; 95% CI 0.5, 1.5) of their grief resolving by the end of the first intervention period.

Of the eight remaining parents in the IOT arm, UG resolved in an additional two parents and returned in one, following the second period where they accessed the education intervention. Of the nine remaining parents in the education arm, UG resolved in an additional one parent and returned in one, following the second period where they accessed the IOT intervention. Those in the IOT arm had a non-significant, reduced adjusted odds (OR 0.91; 95% CI 0.5, 1.6), of their grief resolving by the end of both intervention periods.

### Mental health outcomes (post initial-intervention)

**Figure 2:**
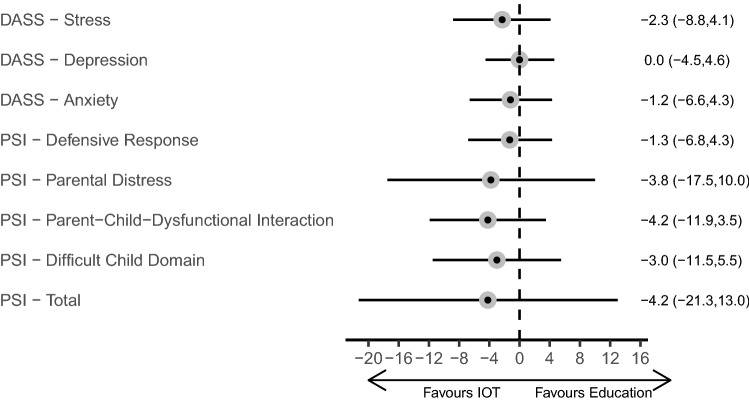
Mean DASS scores across three time-points, by arm.

Mean values for all eight mental health outcomes (scales and totals), were all lower post-invention compared to pre, in both arms (Table 2, Fig. 2). This period one decrease was only statistically significant for DASS-Stress in the IOT arm (− 2.12; 95% CI − 3.57, − 0.68).

**Table 2:** Descriptive statistics for the mental health outcome measures.

Assessment	Subscale	IOT pre	ED pre	IOT post	ED post	IOT Follow-up (following period 2 ED)	ED Follow-up (following period 2 IOT)
n		12	10	8	9	8	7
DASS	Stress	16.7 (8.6)	20.4 (11.1)	12.0 (9.0)**	17.6 (10.5)	10.2 (8.4)**	11.1 (7.7)**
	Depression	8.2 (6.1)	10.4 (10.9)	6.0 (5.5)	8.9 (8.8)	3.0 (2.4)	4.0 (5.3)
	Anxiety	7.5 (5.9)	8.6 (9.4)	2.2 (2.5)	6.4 (8.6)	4.8 (5.1)	5.7 (8.1)
PSI	Defensive response	21.7 (5.3)	21.4 (4.3)	18.1 (3.3)	20.7 (6.2)	17.0 (4.8)**	18.1 (4.2)**
	Parental distress	34.3 (8.2)	33.0 (8.6)	29.2 (7.6)	34.1 (15.1)	27.6 (5.2)	28.9 (4.5)
	Parent–Child-dysfunctional interaction	19.1 (6.5)	23.4 (7.9)	16.4 (4.9)	22.6 (7.5)	17.2 (3.8)	21.1 (4.7)
	Difficult child domain	31.1 (12.0)	31.6 (8.3)	25.0 (6.5)	30.3 (8.2)	24.8 (7.9)	27.7 (5.9)**
	Total	84.5 (22.7)	101.1 (44.6)	70.6 (14.5)	87.0 (27.0)	69.6 (13.1)	77.7 (10.1)**

*IOT* insight oriented psychotherapy, *ED* education.

**p < 0.05 for paired t-test with pre score.

Data presented as mean (sd), or count.

**Figure 3:**
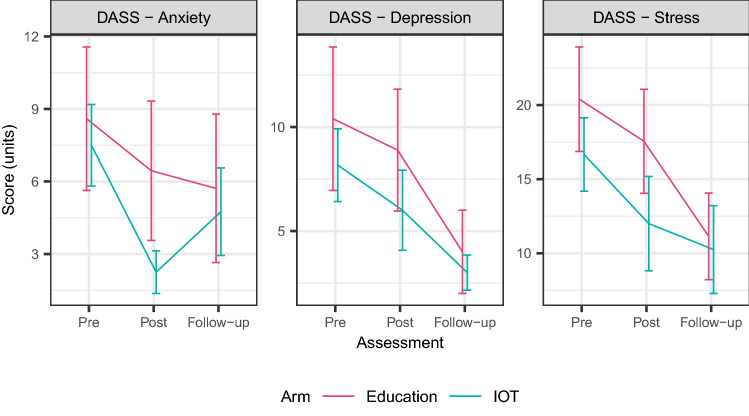
Adjusted mean difference in measures of emotional wellbeing following intervention.

At the end of the first intervention period, the IOT arm had lower (though the confidence intervals included the value of no difference) mean values for seven of the eight mental health outcomes (DASS-Depression being the exception, mean difference estimate 0.0), see Table 3, Fig. 3. The difference estimates ranged in magnitude from − 0.6 (95% CI; − 3.3, 2.1) for DASS-Anxiety to − 4.2 for both PSI—Parent–Child-Dysfunctional Interaction and PSI-Total. Minimally adjusted mean difference estimates are provided in Supplementary Table 1.

**Table 3:** Adjusted mean difference between arms, following the first and second study period and overall.

Assessment	Subscale	Adjusted IOT effect (period 1)^a^	Adjusted IOT effect (period 2)^a^	Adjusted sequence effect (IOT then ED) (periods 1 and 2)^a^
n		17	15	15
DASS	Stress	− 2.3 (− 8.8, 4.1)	1.6 (− 9.6, 12.9)	3.9 (− 6.7, 14.5)
	Depression	0.0 (− 4.5, 4.6)	0.6 (− 5.7, 6.8)	0.3 (− 6.3, 7.0)
	Anxiety	− 1.2 (− 6.6, 4.3)	2.5 (− 4.0, 8.9)	1.1 (− 7.9, 10.0)
PSI	Defensive response	− 1.3 (− 6.8, 4.3)	− 0.7 (− 5.2, 3.9)	0.3 (− 2.7, 3.4)
	Parental distress	− 3.8 (− 17.5, 10.0)	0.0 (− 6.9, 6.9)	0.2 (− 4.0, 4.3)
	Parent–child-dysfunctional interaction	− 4.2 (− 11.9, 3.5)	− 0.3 (− 8.3, 7.6)	− 0.2 (− 3.9, 3.6)
	Difficult child domain	− 3.0 (− 11.5, 5.5)	1.4 (− 9.0, 11.8)	1.6 (− 2.9, 6.0)
	Total	− 4.2 (− 21.3, 13.0)	0.9 (− 13.9, 15.7)	2.9 (− 3.4, 9.3)

*IOT* insight oriented psychotherapy, *ED* education.

^a^Beta coefficient for the between group difference (IOT arm relative to education arm) from a linear regression model, using an ANCOVA framework, adjusted for parents age, duration of grief, gender, and the (baseline) measure at the start of the period being analysed.

### Secondary analysis (follow-up; post alternate-intervention)

Mean values for most mental health outcomes (scales and totals), were lower at the follow-up time-point compared to the post time-point, in both arms (Table 2, Fig. 2); exceptions being DASS-Anxiety and PSI-Parent–Child-Dysfunctional Interaction in the IOT arm, noting that this was following this cohort’s education intervention. None of the post time-point to follow-up time-point decreases were statistically significant, however overall decreases (pre time-point to follow-up time point) were significant for two outcomes (DASS-Stress and PSI-Defensive-Response) in the IOT arm and for four outcomes (DASS-Stress, PSI-Defensive–Response, PSI-Difficult-Child-Domain and PSI-Total) in the ED arm.

At the end of the second intervention period, the IOT arm (who had just received education) had higher (though the confidence intervals included the value of no difference) mean values for six of the eight mental health outcomes (PSI-Defensive Response and PSI-Parent–Child-Dysfunctional Interaction being the exceptions), see Table 3.

The analysis of both intervention periods (with follow-up time-point as the dependent variable, and overall sequence (IOT then education) being the variable of interest) yielded no significant differences for the eight mental health outcomes; four of the eight outcomes had a mean between-sequence difference of < 1.0 unit in magnitude.

## Discussion

Parental unresolved grief (UG) related to their child being diagnosed with CF was highly prevalent amongst parents, and for some this had been present over a decade post diagnosis. Anxiety and stress were more frequently present in parents with UG. IOT and CF education (in either delivery sequence) appeared to mitigate parental grief and resulted in resolution of grief. Whilst increased rates of depression and anxiety and higher levels of stress have been well described in patients with CF and their caregivers^16^, and UG related to other chronic and permanent conditions has been described^17–21^, to our knowledge this is the first study to investigate UG related to a diagnosis of CF and also the first clinical trial to investigate methods to facilitate resolution of such grief.

In our study population grief associated with learning of a child’s CF diagnosis remained unresolved for up to 14 years; this extended duration of grief is similar to grief related to loss of a close family member^22,23^, which is also associated with adverse mental health outcomes, insecure attachment^24^, and has been demonstrated to associate with adverse effects on the child’s developing sense of self^10^.

The high percentage of resolution of grief seen following the relatively brief interventions applied in our study is encouraging, and may have implications for other conditions. For example, substantial levels of UG has been reported in parents of children with other permanent conditions such as Down syndrome, autism spectrum disorders, cerebral palsy, epilepsy, and phenylketonuria^17–21^.

The signal for resolution of grief was supported by strong signals for improvement of comorbidities. All parents with both unresolved grief and depressive symptoms at screening reported a decrease in depressive symptoms once both interventions were completed. Also, 91% of parents with both unresolved grief and anxiety at screening experienced a reduction in anxiety symptoms once both interventions were completed. Increased rates of depression and anxiety and higher levels of stress have been well described in caregivers of children with CF^17–21^. Therefore, improvements in both anxiety and stress levels following resolution of grief was encouraging. It must be acknowledged that untangling the contribution of each individual treatment versus the combination of treatments (full sequence of intervention) in regards to their individual contribution to these improvements is challenging in this setting; it is likely a larger sample and a different study design would be required to fully address this question.

Overall, the interventions examined in this study were feasible to implement within the clinical setting; the relatively high retention rate of participants (who are managing a chronic condition) across the duration of the study suggests either intervention could be adopted into clinical care with relative ease.

The study had a number of limitations. Firstly, the sample size was limited. However, results clearly showed that grief that had been present for years resolved for multiple participants following the relatively brief study period. For some participants the limited intervention may not have been enough to fully explore the depth of their feelings regarding their child’s diagnosis and result in resolution of grief, hence the resolution of grief seen with the limited intervention was encouraging. A multi-centre study will be required to enrol sufficient numbers of participants to obtain more definitive results. Secondly, study participants were mostly mothers. The implications of our findings to fathers therefore require further study. Thirdly, only one certified RDI coder was used to analyse the data to make the UG diagnosis. Despite this, the reduction in ‘coder assessed UG’ coincided with a substantial reduction in mental wellbeing indicators, supporting the study’s ability to objectively assess change following intervention.

The present study also demonstrated that revisiting CF related education proved to assist parents to resolve UG. All participants would have received at least a 2 day block of CF education shortly after their child’s diagnosis, been provided with appropriate CF health literature and referred to appropriate websites e.g. CF Foundation. Clearly, offering CF related education to parents shortly after diagnosis (i.e. during a time of high stress, shock and disbelief) may not be conducive towards the information being taken on board and processed, and might result in distortion of information. Providing parents with an opportunity to revisit CF education after a period of adjustment may enable parents to obtain clarity and reprocess information more accurately.

The measurement of UG using the RDI is both time consuming and costly, it requires clinicians to be trained in the administration and scoring of this complex measure. A recent study by Sher-Censor et al.^25^ examines a less costly and more time-effective self-reported measure of parental resolution, the Reaction to Diagnosis Questionnaire (RDQ), which assesses parental resolution in relation to the child’s diagnosis and looks to show promise.

In summary, UG is highly prevalent in parents of children with CF, can last over many years and is associated with mental health comorbidities; however, it can potentially be resolved with education and/or psychotherapy. Results suggest the need to investigate how to improve practices around CF diagnosis and incorporate grief identification and appropriately targeted intervention for parents. These results have potential application for other chronic conditions diagnosed in infancy.

## Supplementary Information

Supplementary Information.
