# Supplementary material for: A pilot study of disease related education and psychotherapeutic support for unresolved grief in parents of children with CF
Source: Sci Rep. 2022 Apr 6;12:5746. doi: 10.1038/s41598-022-09463-8 (PMC8987037; doi:10.1038/s41598-022-09463-8)
Supplement: Supplementary file 1 — Supplementary Information. [file 41598_2022_9463_MOESM1_ESM.pdf]

A pilot study of disease related education and psychotherapeutic support for unresolved grief in parents of children with CF

André Schultz[1,2,3], Andrea Barrett[3], Elizabeth Balding[1], Wesley Billingham[1], Cindy Branch-Smith[1], Zubin Grover[3], Gisele Yikilmaz[1], Crystal Bourke[3], Julie Depiazzi[3], Nicole Sander[3], Juliet Foster[4], Matthew Cooper[1], Florian Zepf[1,2,5,6]

[1]*Wal-yan Respiratory Research Centre, Telethon Kids Institute, University of Western Australia, Perth, Australia*

[2]*Division of Paediatrics and Child Health, Faculty of Medicine, University of Western Australia, Perth, Australia*

[3]*Perth Children's Hospital, Perth, Western Australia, Australia*

[4]*Woolcock Institute of Medical Research*

[5]*Department of Child and Adolescent Psychiatry, Psychosomatic Medicine and Psychotherapy, Jena University Hospital, Friedrich Schiller University Jena, Germany*

[6]*Centre and Discipline of Child and Adolescent Psychiatry, Psychosomatics and Psychotherapy, Division of Psychiatry & Clinical Neurosciences, School of Medicine, Faculty of Health and Medical Sciences, University of Western Australia, Perth, Australia*

Corresponding author: André Schultz (andre.schultz@health.wa.gov.au)

ONLINE RESOURCE 1

Description of the Reaction to Diagnosis Interview

The Reaction to Diagnosis Interview (RDI) is a structured interview which consists of 5 (or 6 if needed) open ended question which takes approximately 10-15 minutes to complete. It was developed by Pianta and Marvin to examine parent's resolution of the loss/trauma associated with learning of their child's diagnosis of disability and/or chronic illness. The questions are designed to probe parents about their thoughts and feelings associated with their child's medical difficulties, their experiences leading up to the diagnosis and how their thoughts and feelings have changed over time.

The RDI coder determines a parent's resolved/unresolved classification by analysing the video material of each parent repossess to the interview questions.

Parents are deemed to be resolved when responses have the following features; recognition of change since the diagnosis, there is an assertion of moving on with life, have suspended searching for a reason for the diagnosis, have an accurate representation of their child's abilities and have a balanced view regarding some of the benefits of their child's diagnosis 'e.g., we spend more time together as a result of daily treatment"

Parents classified as unresolved are disoriented in their thought processes and their responses have the following indicators; cognitive distortion about the reality of their child's diagnosis (e.g. unrealistic expectations), ongoing search for a reason for the diagnosis, continuing to be "stuck in the past: with no evidence of change over time, confusion and mental disorganisation about the diagnosis, show signs of being cut off from the experience of the diagnosis, are in denial that the diagnosis has affected their lives and have displaced their reactions to the diagnosis by focusing away from their child e.g. excessive focus on fund raising at the expense of time with their child. The presence of these indicators prevents the parent from focusing on their child's emotional needs and behavioural cues resulting in parenting challenges (RDI manual p7-12).
The coder not only analyses the narrative, but also the parent's affect, and their interactions with the interviewer.

The RDI has an interrater agreement ranging from 84%- 96% in reliability[1]. Mothers' resolution of diagnosis is strongly related to their child's attachment classification. Children of parents who were classified as "resolved" are more likely to have a secure attachment to their parent. In one study, 81% of mothers who were classified as unresolved, were found to have children with an insecure attachment[2].

Poslawsky et al[3] found that stability of classification (resolved vs. unresolved) was maintained over time.

Insight Oriented Therapy (IOT)

Having insight into one's difficulties has been considered an important mechanism for psychological change. Insight oriented therapy can be defined a therapeutic process in which psychological change occurs as a patient develops greater self-understanding (insight) and affective awareness of themselves[4]. Therapy occurs in the context of a therapeutic relationship which aims to help the patient develop an understanding of the life events and psychological factors that have contributed to their current difficulties. Insight is an important factor in psychotherapy[5]. By gaining insight, patients not only become aware of how past events and experiences are connected to their current psychological difficulties but also develop awareness of how they themselves contribute to the recurrence of these difficulties[5]. An important aim of psychotherapy is enduring, long lasting change. Both improved insight and self -awareness are the mechanisms for the long- term effects of psychotherapy[6].

As this study focused on unresolved parental grief, a central focus of therapeutic intervention was to discuss parent's grief associated with their child's CF diagnosis. For parents, one of the most challenging experiences a parent can face is coming to terms with their child's illness as these thoughts and feelings create agonising emotions[7] where parents are caught between the tasks of caring for their unwell living child, leaving little or no time to grieve the child they dreamt of, the idealized child. Consequently, as the task of mourning becomes repressed, parents are left in a state of chronic mourning to describe the lifelong depression experienced by these parents[7].

In this study, psychotherapy was limited to 5 sessions. The therapy focused on a number of events including:

1. The parent's experience of hearing the news of the diagnosis
2. The parent's experience of their child's diagnosis on a day to day basis and long term
3. Identifying previous knowledge of CF and the impact of this on their own understanding of their child's illness
4. How the parent's own childhood experiences have influenced their parenting
5. Current parent-child relationships
6. The parent's feelings of grief and loss associated with their child's diagnosis
7. How the parents and the family manage the burden of care in caring for their child.
8. Reflections on the parent's emotional experiences about the future for their child

This study provided an opportunity for parents to discuss their experiences in the context of their child's diagnosis with an experienced clinical psychologist. For some study participants this was the first time they had spoken about these experiences and the impact this had on their lives. Through the process of therapy, parents gained insight through greater self-awareness and self-understanding of the grief and loss they felt about their child's diagnosis. Through IOT parents were helped to resolve the grief associated with their child's diagnosis of cystic fibrosis.

References

1 Pianta RC, Marvin RS, Britner PA, Borowitz KC. Mothers' resolution of their children's diagnosis: Organized patterns of caregiving representations. Infant Mental Health Journal. 1996; **17**: 239-56.

2 Marvin RS, Pianta RC. Mothers' reactions to their child's diagnosis: relations with security of attachment. J Clin Child Psychol. 1996; **35**.

3 Poslawsky IE, Naber FB, Van Daalen E, Van Engeland H. Parental reaction to early diagnosis of their children's autism spectrum disorder: an exploratory study. Child Psychiatry Hum Dev. 2014; **45**: 294-305.

4 Strachey J. The nature of the therapeutic action of psycho-analysis. 1934. J Psychother Pract Res. 1999; **8**: 66-82; discussion 64-5.

5 Jennissen S, Huber J, Ehrenthal JC, Schauenburg H, Dinger U. Association Between Insight and Outcome of Psychotherapy: Systematic Review and Meta-Analysis. Am J Psychiatry. 2018; **175**: 961-9.

6 Hoglend P, Hagtvet K. Change mechanisms in psychotherapy: Both improved insight and improved affective awareness are necessary. J Consult Clin Psychol. 2019; **87**: 332-44.

7 Erel-Brodsky H. Ghosts in the nursery: the secret thoughts of a sick child's parents. Am J Psychother. 2014; **68**: 81-102.

ONLINE RESOURCE 2

Amendments to the protocol occurred but we believe that these amendments did not change the results of the study.

Amendments were as follows:

a. The main study aim of the research was, from the outset, focused on (parental) unresolved grief related to the diagnosis of CF in a child. At the outset of the study, we were realistically concerned that there may not be sufficient numbers of parents of children with CF who are experiencing unresolved grief available for recruitment in the study (acknowledging we run the state-wide multidisciplinary care centre for all children with CF in Western Australia; population ~2.6milion; paediatric CF population ~200). Therefore, in the initial protocol drafts, we incorporated measures of anxiety and depression as the primary measure as opposed to unresolved grief. Early in recruitment, prior to the randomisation of anyone without unresolved grief, we realised that unresolved grief was sufficiently prevalent to limit the study to only include parents with unresolved grief (as originally intended). The protocol was therefore amended to only randomise parents with unresolved grief (with or without associated anxiety or depression) and use the RDI as primary outcome measure. In summary, while we initially gave ourselves the option to randomise participants who did not have unresolved grief, we never used that option. Hence, the protocol is described to reflect the actual process that occurred.

b. The study was originally designed to include parents of children 6 months – 10 years of age but was later amended to include parents of older children.

ONLINE RESOURCE 3

A) OUTLINE OF RESPIRATORY COMPONENT OF EDUCATIONAL INTERVENTION (conducted by respiratory physician)

- What is cystic fibrosis?
- Basic genetics and inheritance explained
- Details about organs affected including specifically lung, gut, pancreas.
- Basic CF related airway surface physiology linked to essentials about management options, with focus on the respiratory system
    - Normal muco-ciliary clearance
    - Abnormal chloride and sodium transport in CF that results in dehydrated airway surface liquid and abnormal muco-ciliary clearance
        - Chest physiotherapy airway clearance
        - Hypertonic saline nebulisations
        - Inhaled mannitol
    - Predisposition to infections on the airway surface
        - Use of antibiotics in exacerbations and other situations
    - Dysregulated neutrophilic inflammation
        - Azithromycin as an anti-inflammatory agent
    - How the CFTR gene is expressed
    - How CFTR gene expression can go wrong in CF
        - CFTR modulator therapies
        - More hope for the future: briefly what has been achieved with CF gene therapy to-date
- Overview of support provided by hospital. The CF multidisciplinary team.
- Overview of support provided by community. Local and national NGOs
- Prognosis and what to expect –a optimistic approach
- Time for questions

B) OUTLINE OF GASTROENTEROLOGY COMPONENT OF EDUCATIONAL INTERVENTION (conducted by gastroenterologist)

- Interactive, simple, visual based educational slides in a non-rushed setting.
- Time allocated 45 minutes, while prepared material is delivered through power point but more emphasis is given on answering parent’s questions. Following issues are covered.
    - How does a normal gastrointestinal system work?
    - How is gastrointestinal system affected in cystic fibrosis?
    - Need for nutritional surveillance?
    - What to expect in GI CF clinic appointments?
    - Overview of support provided by specialist gastroenterologist?

- Time for questions

C) OUTLINE OF NUTRITIONAL COMPONENT OF EDUCATIONAL INTERVENTION (conducted by dietician with expertise in CF)

Overview of nutritional aspects of CF

Good nutritional status in Cystic Fibrosis is imperative to achieve normal growth and development. As a result of the pathophysiology of cystic fibrosis, there are numerous barriers to achieving optimal nutrition. The prevalence of malnutrition in CF is common and generally results from a combination of reduced oral intake, increased energy expenditure and fat malabsorption which results in negative energy balance and weight loss/failure to gain optimal weight.

Common barriers to achieving optimal nutritional status in CF include the following;

- Increased energy expenditure
- Deranged bowel actions
  - Nutrient malabsorption
  - Cramping, abdominal pain, abdominal distension, constipation, steatorrhoea
- Poor oral intake
  - Poor appetite, early satiety, fussy eating
  - Illness & infection (raised inflammatory markers related to anorexia)
- Frequent coughing resulting in nausea and vomiting
- Additional conditions of CF
  - Reflux (GOR)
  - CF-related diabetes (CFRD)
  - Distal intestinal obstruction syndrome (DIOS)
  - CF-related liver disease (CFLD)
  - CF-related osteoporosis
  - Anaemia of chronic disease
- Behavioural/psychological problems

The role of the dietitian

- To achieve normal growth and development
- To optimise absorption of nutrients by appropriately using Pancreatic Enzyme Replacement Therapy (PERT)
- To replace electrolytes lost through increased sweat levels.
- To educate patients and their parents regarding other factors associated with CF (e.g. fat soluble vitamin deficiency, altered gastric motility, impaired glucose tolerance / diabetes, and reduced bone mineral density)

CF diet

- High energy, high salt
- Shift from high fat to high energy (including healthy fats and high protein)

The importance of PERT

- What is PERT?
- How to use it/how to administer it
- Fat counting and dosing including resources to support this process e.g. books, phone apps

Why is salt important?

- Recap of pathophysiology of CF and increased salt losses
- How to use it/how to administer e.g. add salt to food, salt liquid, salt tablets, electrolyte/sports drinks
- Winter vs Summer dosing
- Increased requirements with exercise and activity
- Signs and symptoms of inadequate salt

Fat-soluble vitamins

- Reiterate pathology of CF in the context of fat malabsorption
- How to use it/how to administer e.g. crushed, swallow whole, syringe
- Prescription for age

D) OUTLINE OF AIRWAY CLEARANCE PHYSIOTHERAPY COMPONENT OF EDUCATIONAL INTERVENTION

Why is physiotherapy needed for children with cystic fibrosis?

Chest physiotherapy treatment is able to:

- Clear Mucus from the lungs to help prevent chest infections
- Remove excess mucus when an infection is present
- Help the lungs work normally

Chest Physiotherapy needs to:

- Be ongoing even when no symptoms are obvious
- Start as soon as possible after diagnosis of cystic fibrosis

How do the lungs work?

- The lung
- The airways
- The alveoli
- The muscles of breathing

What is involved in chest physiotherapy?

- Mechanism of clearance
- Nebulised Solutions
- Positioning
- Technique
- FET
- Exercise and activity

How may chest infections be identified?

- Cough
- Mucus
- Changes in breathing.
- Increased tiredness or reduced activity
- Decreased appetite and/or weight loss
- Increases irritability
- Fever

How will physiotherapy treatments fit into our daily routine?

How will Physio change as my child grow?

E) OUTLINE OF NURSING COMPONENT OF EDUCATIONAL INTERVENTION (conducted by CF nurse)

This session was mostly scheduled as the final in the series of the five hour-long education sessions

The CF nurse would aim to individualise education given and focus on on perceived gaps knowledge

Topics covered:

- Brief overview of CF
- Basic understanding CF physiology, disease and genetics.
- Discussion about practical aspects of CF medications, ordering, dispensing and storage
- Discussion on administration of medications (practical aspects)
- Discussion about how CF can affect the family including family dynamics
- Discussion about prognosis (a positive approach)

Supp Tab. 1 – Minimally adjusted mean difference between arms, following the first and second study period and overall

Assessment	Subscale	Adjusted IOT[a] effect (period 1) *	Adjusted IOT effect (period 2) *	Adjusted sequence effect (IOT then ED[b]) (periods 1 and 2) *
**n**		*17*	*15*	*15*
**DASS**	*Stress*	-1.4 (-7.1,4.3)	1.5 (-6.1,9.1)	2.7 (-4.8,10.1)
	Depression	-1.2 (-4.9,2.4)	-0.2 (-4.3,3.9)	-0.6 (-5.1,3.9)
	Anxiety	-2.0 (-6.7,2.7)	2.7 (-2.5,7.9)	1.0 (-5.2,7.2)
**PSI**	*Defensive Response*	-2.6 (-7.2,1.9)	-0.2 (-4.2,3.8)	-1.0 (-4.7,2.6)
	Parental Distress	-5.8 (-17.1,5.6)	-0.1 (-5.7,5.5)	-1.4 (-6.0,3.2)
	Parent-Child-Dysfunctional Interaction	-2.9 (-8.7,2.9)	-0.7 (-5.9,4.5)	-1.1 (-5.2,2.9)
	Difficult Child Domain	-2.9 (-9.5,3.7)	1.2 (-5.8,8.2)	0.9 (-3.5,5.2)
	Total	-8.0 (-21.5,5.4)	-0.4 (-12.8,12.0)	-2.9 (-12.6,6.7)

[a]IOT - insight oriented psychotherapy; [b]ED - education

* Beta coefficient for the between group difference (IOT arm relative to education arm) from a linear regression model, using an ANCOVA framework, adjusted only for the (baseline) measure at the start of the period being analysed
